# Bilateral back squat strength is increased during a 3-week undulating resistance training program with and without variable resistance in DIII collegiate football players

**DOI:** 10.7717/peerj.12189

**Published:** 2021-09-24

**Authors:** Jason Sawyer, Paul Higgins, Paul A. Cacolice, Troy Doming

**Affiliations:** 1 Health and Physical Education Department, Rhode Island College, Providence, RI, United States of America; 2 Department of Movement Science, Sport and Leisure Studies, Westfield State College, Westfield, MA, United States of America; 3 Science Department, Van Sickle Academy, Springfield, MA, United States of America

**Keywords:** Variable resistance training, Accommodating resistance, Undulating periodization

## Abstract

**Background:**

Optimizing training adaptations is of the utmost importance for the strength and conditioning professional. The pre-season of any sport is particularly important to ensure preparedness of the athletes. In DIII Collegiate Football pre-season consists of approximately 3 weeks. The abbreviated time of the pre-season increases the importance of optimizing training using safe methods, including alternative loading strategies. The purpose of the current study was to determine if a 3-week variable resistance training VRT during an undulating (UL) resistance training program elicited a greater increase in back squat strength compared to traditional loading methods.

**Methods and Materials:**

Forty DIII Football players (age range: 18–25 years) participated in a 3-week UL bilateral back squat (BBS) program. Both groups performed the BBS 3 times per week with a minimum of 24 hours between exercise sessions. The control group (C) (*n* = 20) (height = 182.3 + 5.1 cm, body mass: pre = 102.8 ± 17.7 kg, post = 104.1 ± 17.8 kg) used traditional loading methods (*i.e.*, Olympic weights only) and the experimental group (E) (*n* = 20) (height = 180.7 ± 8.0 cm, body mass: pre = 100.3 ± 27.1 kg, post = 101.0 ± 27.7 kg) used traditional loading methods and variable resistance (*i.e.*, resistance bands). The variable resistance accounted for approximately 20% of the total resistance while 80% of the resistance was supplied by traditional loading methods.

**Results:**

When all data was pooled, subjects had a significant increase (*p* < 0.05) in 1-RM BBS from pre (154.2 + 26.1 kg) to post (166.8 + 26.2 kg), with a percent increase of 8.13% at the completion of the 3-week training program. There was no significant difference (*p* > 0.05) between the C and E groups for muscular strength, muscular power, or vertical jump. Volume-loads were not significantly (*p* > 0.05) different between groups for any of the weeks (C: Week 1 = 858.1 + 101.3, Week 2 = 588.6 + 69.2, Week 3 = 332.5 + 38.9, Total = 1179.2 + 209.4 *vs*. E: Week 1 = 835.2 + 179.7, Week 2 = 572.2 + 123.4, Week 3 = 323.5 + 68.8, Total = 1730.9 + 371.8) or for the pre-season as a whole.

**Conclusion:**

A traditional UL resistance training program and training program with variable resistance are both effective methods at increasing back squat strength during 3 weeks of training. Resistance band variable resistance (VR) does not enhance training effects within a 3-week mesocycle greater than traditional resistance.

## Introduction

Lower body strength (LBS) has a significant impact on various athletic performance variables including linear velocity, change of direction speed, rate of force development (RFD) and prevention of injury (Gabbett et al., 2009; Marques & Izquierdo, 2014; Suchomel, Nimphius & Stone, 2016; Thomas et al., 2016). Because of the importance of LBS on a variety of performance characteristics, the most efficient ways for increasing LBS are constantly being examined. The bilateral back squat (BBS) is used to increase LBS and is theorized to improve the components of sport (*e.g.*, agility, speed, vertical power). Recently, researchers determined that the addition of VRT increased BBS squat by more than 2.5% compared to traditional loading in collegiate soccer players after 6 weeks of training (Katushabe & Kramer, 2020).

During maximal isotonic, multi-joint resistance exercises, such as the BBS, performance is limited by the ability to produce force at the range of motion (ROM) with the least mechanical advantage (Galpin et al., 2015). The ROM where an individual is weakest is often termed the “sticking point.” Because of this limitation, methods to increase force production throughout the entire ROM are continually being investigated. The ability to produce greater muscular force requirements throughout the ROM may lead to a greater training response.

Variable resistance training (VRT) utilizes equipment (*e.g.*, resistance bands and chains) that allow for a progressive resistance force throughout the entire ROM of an exercise (Anderson, Sforzo & Sigg, 2008). The use of this type of equipment was popularized by powerlifters and other strength athletes attempting to increase maximal strength (Swinton et al., 2009). Anecdotally, athletes and coaches have reported that VRT is a cost effective way of increasing strength and performance (Joy et al., 2016).

Recently, athletes and strength and conditioning coaches have begun to use VRT to elicit a post-activation potentiation (PAP) effect. Seitz, Mina & Haff (2016) examined the effectiveness of VRT assisted back squat on PAP. The authors (Seitz, Mina & Haff, 2016) determined that standing broad jump performance was significantly increased by 1.7% after performing back squats with resistance bands. Similarly, Wyland, Van Dorin & Reyes (2015) determined that multiple sets of bilateral back squats performed with VRT were effective for eliciting PAP and improved short-sprint performance of 9.1 m (10 yards) in length.

VRT causes alterations in the kinematics and kinetics of various upper and lower body exercises, (Galpin et al., 2015; García-López et al., 2016; Rhea, Kenn & Dermody, 2009; Saeterbakken, Andersen & Van Den Tillaar, 2016; Stevenson et al., 2010) by altering the resistance throughout the exercise movement. These alterations may make these exercises more effective at increasing muscular strength. Saeterbakken, Andersen & Van Den Tillaar (2016) examined the effects of VRT on kinematics and muscle activation during the BBS and determined that total resistance with VRT was 5% greater at the sticking point and 13% greater after the sticking point compared to traditional free-weights. Because of this, the authors (Saeterbakken, Andersen & Van Den Tillaar, 2016) suggest using VR in addition to traditional loading to enhance heavy resistance training. When using VRT, Andersen et al. (2016) determined that there was increased quadriceps and hamstring activation during the concentric contraction phase of the back squat compared to constant loading methods. Furthermore, the authors (Andersen et al., 2016) reported that there was a dose-dependent response, suggesting that using resistance bands for a higher percentage (*i.e.,* >35%) of the total load may be advantageous. Stevenson et al. (2010) determined the peak RFD was acutely increased by 123.9 N m^−1^ during the back squat by adding variable resistance. Researchers (Anderson, Sforzo & Sigg, 2008; Rhea, Kenn & Dermody, 2009; Rivière et al., 2016) have determined significantly greater strength and power gains could occur in the upper and lower body using VRT when compared to free-weight-only loading methods.

NCAA DIII represents the largest division, yet research regarding ways to optimize resistance training is lacking. The length of the pre-season in DIII collegiate athletics may be relatively short (*i.e.,* 3 weeks) to comply with NCAA rules and regulations. The abbreviated pre-season poses significant obstacles for strength and conditioning professionals attempting to prepare athletes for in-season competition. Research utilizing shorter mesocycles need to be completed to determine the minimum length for improvements in strength and conditioning. In one such study, researchers (Argus et al., 2010) determined that a 4-week resistance training mesocycle increased upper- and lower-body strength, fat-free mass, and decreased fat mass in elite rugby players. These findings indicate that improving strength and body composition may occur with short (*i.e.,* <4 weeks) resistance training programs. The effects of short-term resistance training mesocycle should be determined in other cohorts including those participating in contact sports, such as American Football.

VRT is effective for eliciting an accretion in muscular strength when combined with free-weights (Anderson, Sforzo & Sigg, 2008; Rhea, Kenn & Dermody, 2009; Rivière et al., 2016; Wyland, Van Dorin & Reyes, 2015). However, the minimum dose of VRT has yet to be determined. The purpose of this research study was to determine if the addition of variable resistance during the BBS exercise would cause greater increases in lower body strength and power compared to traditional loading methods (*i.e.,* Olympic plates) during a short-term 3-week, pre-season undulating (UL) training program in NCAA DIII athletes. It was theorized that during the 3-week training program, the use of VR in combination with free-weight resistance would cause a greater increase in back squat strength and power output than free-weights alone.

## Materials & Methods

Forty-three NCAA DIII Football players were recruited for participation in a 3-week UL resistance training program, with forty of the subjects completing the study (*n* = 40). All subjects signed an informed consent with the understanding that subjects could withdraw from participation at any point in the study. Participants were required to complete a medical history questionnaire, which was reviewed by a licensed physical therapist to ensure safe participation. The study was approved by the Institutional Review Board (IRB) of Westfield State University. This project was conducted as part of a voluntary pre-season conditioning program for the team. The original study protocol approved by the IRB listed a 3 repetition maximum bilateral back squat (3-RM BBS) and a linear periodization program be followed. However, during testing a 1-RM BBS was performed and an undulating program was prescribed. These changes in protocol were approved by the IRB in addendum after the completion of the study.

### Procedures

Participants were randomized into two groups, Control, (*n* = 20) and Experimental (*n* = 20). The study consisted of a pre-test, nine training sessions, and a post-test session. Pre-testing was administered to determine baseline values for height, body mass, vertical jump (VJ) (Vertec, Jump, USA), power output (GymAware, Kinetic Performance Technology) and 1-repetition maximum BBS (1RM-BBS). Leard et al. (2007) determined that the Vertec had a high correlation (Pearson’s *r* = 0.91) with 3-motion camera analysis, a criterion method of determining vertical jump height. Researchers (O’Donnell et al., 2018) reported a high correlation for vertical jump height when using the GymAware force transducer (Pearson’s *r* = 0.90) when compared to a force plate and a mean intraclass correlation of 0.70.

Baseline testing occurred a minimum of 5 days prior to the start of the experimental protocol. The training program consisted of three BBS sessions per week for three weeks in which participants used loads as a percentage of their respective 1RM-BBS. The intensity for each workout was undulated from heavy/light/medium, respectively. The sets, repetitions and percentages used for the BBS are listed in Table 1. BBS was performed first during each workout with other compound and accessory exercises completed thereafter. The compound and accessory exercises included bench press, hang power cleans, shoulder press, pull-ups, bicep curls, triceps extensions, and core exercises (plank and other variations) and were programed according to NSCA guidelines (Haff & Triplett, 2016). The C group performed the BBS exercise using free-weights only, while the E group performed sets with free weights and 20% of the training load applied by variable resistance via resistance bands (EliteFTS Inc.) attached to the barbell.

**Table 1:** Sets, repetitions, and percentages used for BBS during the 3-week program.

Workout number	Sets	Repetitions	Percentages (%)
1	5	7	78
2	5	3	50
3	5	5	80
4	5	4	83
5	5	3	55
6	5	3	85
7	5	2	90
8	5	1	93
9	5	3	50

Band tension was determined using a digital bow scale (Superior Balance, 110 lbs. Digital Bow Scale, OK, USA). The use of a bow scale was previously used by Anderson, Sforzo & Sigg (2008) and estimated tension to the nearest 0.2 kg. Band tension was determined while the band was stretched at 176.2 cm. This distance was chosen because it represented the average length of the band when using the band attachments on a standard squat rack (Pro Series Double-Sided Half Cage, Legend Fitness, TN, USA). The post-test consisted of VJ and 1-RM BBS and was performed three days after the completion of the training program. No special incentives were given to the subjects. All testing and training sessions were supervised for safe technique by a certified strength and conditioning coach.

Upon completion of the study, volume loads for both groups were calculated using the following formula: volume load = number of repetition multiplied by the weight (kg) lifted (*i.e.,* [repetitions (no.) × external load (kg)]. This formula has been used by previous researchers (Peterson et al., 2011). Volume load was calculated for week 1, 2, and 3 and for the entirety of the resistance training program.

### Statistical analysis

When equipment outcome was quantified in Imperial units, values were converted to International Units. Data were then entered into commercially available software (SPSS v26, IBM, Armonk, NY). To assess if there were demographic differences between the C and E groups, a One-Way ANOVA was utilized to explore for body mass and height mean differences between groups. A One-Way ANOVA was utilized to explore differences in volume load between groups during each week, and for the entire three-week pre-season as a whole. To determine any outcome training difference from the intervention of the resistance bands, a One-Way ANOVA was performed to determine associations for VJ height, power output, and 1-RM BBS between C and E groups. To assess the effectiveness of the undulating periodization program on the sample in such a short time frame as a NCAA DIII pre-season, a One-Way ANOVA was performed to determine associations for VJ height, power output, and 1-RM BBS for pre- and post-testing for the entire sample. A Cohen’s *d* was then generated reflecting the effect size of any statistically significant associations at *p* ≤ .05. Utilizing any generated Cohen’s d effect size, alpha error probability, and total sample size, post-hoc statistical power was calculated utilizing statistical freeware (G*Power, v 3.1.9.6, Düsseldorf, Germany).

## Results

Forty (*n* = 40) subjects completed the study. Subjects that missed two or more training session were excluded from the data set. Compliance for the C and E group was 95.0% and 93.8%, respectively. Initially forty-three subjects were recruited however, two subjects were unable to complete the study due to injuries unrelated to the research and one subject was unable to attend the post-testing session resulting in the *n* = 40.

The C and E groups were not significantly (*p* > 0.05) different for height and body mass pre- or post- training. Descriptive statistics for C and E groups are listed in Table 2.

**Table 2:** Descriptive Statistics for Participants (mean ± standard deviation).

	Control		Experimental	
	Pre	Post	Pre	Post
Body Mass (Kg)	102.8 ± 17.7	104.1 ± 17.8	100.3 ± 27.1	101.0 ± 27.7
Height (cm)	182.3 ± 5.1		180.7 ± 8.0	

**Notes.**

* Significant difference between Control and Experimental Groups (*p* ≤ .05).

### 1-RM BBS:

There was no significant interaction between the C (pre = 156.3 ± 18.5 kg; post = 167.7 ± 17.9 kg) and E (pre = 152.4 ± 32.7 kg; post = 165.8 ± 32.8 kg) groups for the 1-RM BBS. When the groups were pooled, 1-RM BBS back squat increased significantly (*F* = 6.15, *p* = .018) pre to post for both groups (pre 154.2 ± 32.7 kg *vs.* 165.8 ± 26.2 kg). Descriptive statistics for 1-RM BBS, VJ height, and power output are listed in Table 3. The Effect Size of this interaction was calculated as Cohen’s *d* = .476.

**Table 3:** Descriptive Statistics of mean values and standard deviations for VJ Height, Power Output, 1RM BBS (mean ± standard deviation).

	Control		Experimental	
	Pre	Post	Pre	Post
VJ (cm)^a^	61.7 ± 12.0	59.8 ± 10.9	58.9 ± 11.1	59.3 ± 10.2
Power Output (Watts)	7196.8 ± 1746.5	7805.6 ± 1117.9	7040.2 ± 1920.5	7567.3 ± 2123.4
1-RM BBS (Kg)^b^	156.3 ± 18.5	167.7 ± 17.9	152.4 ± 32.7	165.8 ± 32.8

**Notes.**

* Significant difference between control and experimental groups ( *p* ≤ .05).

a Vertical Jump.

b One-repetition maximum for barbell back squat.

### Vertical jump height:

There was no significant (*p* > 0.05) interaction in VJ height pre *vs* post for either the C (pre = 61.7 ±  12.0 cm; post = 59.8 ± 10.9cm) or E (pre = 58.9 ±  11.1 cm; post = 59.3 ± 10.2cm) group. There was no significant (*p* > 0.05) difference between the groups for VJ height.

### Power output:

There was no significant (*p* > 0.05) interaction for power output pre *vs* post for either the C (pre = 7196.8 ± 1746.5 W; post = 7805.6 ± 1117.9 W) or E (pre = 7040.2 ± 1920.5 W; post = 7567.3 ± 2123.4 W) group. There was no significant (*p* > 0.05) difference between the groups for power output.

### Volume-load

Volume-loads were not significantly (*p* > 0.05) different between groups for any of the weeks (C: Week 1 = 858.1 ± 101.3, Week 2 = 588.6 ± 69.2, Week 3 = 332.5 ± 38.9, Total = 1179.2 ± 209.4 *vs.* E: Week 1 = 835.2 ± 179.7, Week 2 = 572.2 ± 123.4, Week 3 = 323.5 ± 68.8, Total = 1730.9 ± 371.8) or for the pre-season as a whole.

### Statistical power

Utilizing the Effect Size from the 1-RM BBS, the calculated statistical power (1- *β*) was .905. As only one mean group difference from our investigation was statistically significant (1RM BBS), only one Cohen’s d was calculated, as was one post-hoc statistical power value.

## Discussion

The minimal dosage in order to increase strength using VRT has yet to be determined. Moreover, given the brevity of the pre-season in NCAA DIII athletics, determining the effects of short-term resistance training program is vital to prepare athletes for in-season play. The primary finding of the current research was that 3 weeks of VRT did not produce greater gains in strength and power any more than traditional loading methods. A secondary finding of the current study was that a short-term UL program was effective at increasing 1-RM BBS.

Previous researchers (Anderson, Sforzo & Sigg, 2008; Rhea, Kenn & Dermody, 2009) reported that VRT was superior for eliciting strength increases compared to traditional loading methods. For example, Anderson, Sforzo & Sigg (2008) examined the effects of VRT on bench press and back strength and power output in male and female DI athletes. The researchers (Anderson, Sforzo & Sigg, 2008) reported that VRT significantly increased upper and lower body strength and power. Specifically, Anderson, Sforzo & Sigg (2008) reported that the VRT group had nearly a three times greater increase in 1-RM BBS strength compared to controls (VRT = 16.5 ± 5.7 kg *vs. C* = 6.8 ± 4.4 kg) after 7 weeks of resistance training with VRT. Rhea, Kenn & Dermody (2009) examined the effect of squat speed with VRT on lower body peak power and force development. Subjects completed a 12-week resistance training program using heavy resistance, light resistance, or light resistance with the addition of VRT. The researchers (Rhea, Kenn & Dermody, 2009) determined that using VRT was superior in eliciting increases in peak force and peak power output in DI athletes compared to traditional loading methods.

The findings of the current study are in contrast to previous research (Anderson, Sforzo & Sigg, 2008; Rhea, Kenn & Dermody, 2009). During the current study there were no significant (*p* > 0.05) differences between C and E groups. A major difference between the studies is the length of the resistance training cycle (7 *vs* 12 *vs* 3 weeks). This may indicate that there is a dose-dependent response for VRT that is greater than 3 weeks. Few studies have examined the effects of VRT during a training intervention lasting 6 weeks (Rivière et al., 2016), and with most lasting greater than 7 weeks (Anderson, Sforzo & Sigg, 2008; Rhea, Kenn & Dermody, 2009).

Another possible cause of the conflicting results between the current study and the broader literature is the cohort that participated in the current study. Seitz, Mina & Haff (2016) reported that the PAP caused by VRT was dependent upon the initial strength level of the subject, with stronger subjects having a greater PAP. The current study included DIII Collegiate Football Players. Fry & Kraemer (1991) have reported that there are significant differences between the various collegiate football divisions for muscular strength and power, with DIII reporting the lowest measures. For example, researchers (Fry & Kraemer, 1991) determined that DIII athletes had a mean maximum bench press and squat that were approximately 16 kg lower than DI counterparts. In addition, the decrements of DIII athletes compared to DI athletes were observed for the power clean, 36.6 meter sprint, and vertical jump heights (Fry & Kraemer, 1991). The training age and/or initial strength measures of the subjects used during the current study may have caused the lack of difference between VRT and traditional loading groups.

Similar to the current study, Loturco et al. (2020) examined the effects of a short-term (4-weeks) traditional free-weight *vs.* variable resistance training on strength, speed, and power performance in elite youth soccer players. The researchers (Loturco et al., 2020) determined that lower body strength and power were increased using free-weights only and VRT. Interestingly, the researchers determined that the addition of variable resistance accelerated the increase in lower body strength and power with the VRT group increasing strength faster within the first two weeks of resistance training compared to the traditional loading group. While the current study did not examine the time course of strength and power gains, it is possible that the VRT group increased strength faster compared to the traditional free-weight group. Loturco et al. (2020) used “moderate” tension resistance bands in the VRT group. Differences in the amount of resistance provided by VRT may have existed, further explaining any differences in results.

Although VRT did not cause a greater increase in back squat strength compared to traditional loading methods, both groups experienced an increase in BBS after completing a 3-week UL program. Researchers (Prestes et al., 2009; Zourdos et al., 2016) have reported that an UL program is effective for increasing strength in trained individuals, and the results of current study support these findings. An UL program is one in which the intensity and volume of exercise is altered between training sessions (*i.e.,* light, moderate, and heavy days).

A weakness of the current study was the length of the resistance training program. Three weeks is considered a short time period for a strength mesocycle. This length of time was specifically chosen because it is the same length of time as the typical pre-season in DIII football. Because of the abbreviated pre-season length in DIII athletics, the most effective ways of increasing strength and preparedness for athletes must be determined. Given the brief duration of the intervention, the magnitude of the effect size of the VRT *vs* traditional loading on various outcomes may have been too small for this study to detect.

Future research should include the addition of more subject groups. Two additional groups of subjects should be used, to allow for a linear periodized without VRT group and linear periodized with VRT group. This would provide a direct comparison of VRT during a linear *vs.* an UL resistance program.

## Conclusion

A short-term, 3-week UL resistance training program is effective at increasing the 1-RM of the BBS. This method of periodization can effectively be used during DIII Collegiate Football pre-season. The addition of VR does not produce greater strength gains in 3 weeks, but may effectively provide an alternative to traditional training methods with similar results.

##  Supplemental Information

10.7717/peerj.12189/supp-1 Supplemental Information 1 Raw Data

## Additional Information and Declarations

### Data Availability

The following information was supplied regarding data availability: The raw measurements are available in the Supplementary File.
